# Grape-Seed Polyphenolic Extract Improves the Eye Phenotype in a *Drosophila* Model of Tauopathy

**DOI:** 10.4061/2010/576357

**Published:** 2010-08-24

**Authors:** Cathie M. Pfleger, Jun Wang, Lauren Friedman, Roselle Vittorino, Lindsay M. Conley, Lap Ho, Hayley C. Fivecoat, Giulio M. Pasinetti

**Affiliations:** ^1^Department of Oncological Sciences, Mount Sinai School of Medicine, One Gustave L. Levy Place, NY 10029, USA; ^2^Department of Neurology, The Mount Sinai School of Medicine, One Gustave L. Levy Place, NY 10029, USA; ^3^James J. Peters Veteran Affairs Medical Center, 130 West Kingsbridge Road, Bronx, NY 10468, USA

## Abstract

*Drosophila* models of tauopathies have been developed by transgenically overexpressing the disease-associated forms of *tau*. In this paper we report for the first time that a recently developed Grape-Seed Polyphenolic Extract (GSPE) improves the eye phenotype of a *Drosophila* eye model of *R406W tau*. GSPE-mediated improvements in this distinct *in vivo* neurodegeneration model for protein misfolding/aggregation suggest that GSPE may have therapeutic value in disorders involving aberrant protein aggregation.

## 1. Introduction

Abnormal protein conformations resulting in misfolded and/or abnormally aggregated protein species are a characteristic feature of several neurodegenerative disorders. For example, the microtubule-associated protein tau adopts abnormal conformations resulting in protein helical filament/immunopositive neurofibrillary tangles (NFTs) and is a characteristic neuropathological feature in Alzheimer's Disease (AD), Pick's disease, progressive supranuclear palsy, and corticobasal ganglionic degeneration (CBD). Tau mutations cause frontotemporal dementia with Parkinsonism- (FTDP-) 17, proving that tau dysfunction can directly promote neurodegeneration [1]. 

*Drosophila* models using the transgenic Gal4/UAS system [2] to overexpress disease-associated aggregation-prone proteins have modeled aspects of tauopathy by overexpressing *R406W *mutant tau [3, 4] and Huntington's disease (HD) by overexpressing* Q93httexon1 *[5–7] (for review, see [8]). Overexpressing *R406W* in cells that form the eye (*ey *>* R406W*) leads to dramatic reduction in or complete absence of the eye. Eyes that do form demonstrate abnormal morphology [4]; for review, see [8]. 

We previously reported that moderate consumption of the red wine, Cabernet Sauvignon (*Vitis vinifera*), prevented abnormal *β*-amyloid (A*β*) oligomerization coincidental with a significant attenuation of spatial memory impairment in a mouse model of AD-type amyloid neuropathology [9]. Most importantly, we identified a polyphenolic compound highly concentrated in grape-seed polyphenolic extract (GSPE) as potentially responsible for the beneficial role of moderate consumption of red wine *(Vitis vinifera*) [10, 11]. 

More recently, Ho et al. reported that, using an *in vitro* aggregation assay, GSPE can significantly inhibit tau peptide Ac(306)VQIVYK(311) aggregation. Moreover, GSPE can also disaggregate preexisting aggregated tau peptides [12]. These results strongly suggest that GSPE might provide beneficial disease-modifying activity in tau-associated neurodegenerative disorders by modulating tau-mediated neuropathologic mechanisms. In this paper, we use the eye phenotype of a Drosophila model of mutant R406W tau to further evaluate the beneficial role of GSPE in tau-mediated neuropathology *in vivo*. We report for the first time that treatment with GSPE significantly benefits *Drosophila* phenotypes carrying mutant tau (*R406W)*, further supporting the potential use *in vivo *of GSPE in abnormal tau aggregation, as previously demonstrated *in vitro* [12].

## 2. Materials and Methods

In this study, MegaNatural grape-seed polyphenolic extract (*GSPE*) was provided by Polyphenolics, Inc. (Madera, CA), as highly purified (>97% total polyphenols) water-soluble polyphenolic preparation from *Vitis vinifera* seeds.

### 2.1. Drosophila Strains

*w*;* eygal4/SM6-TM6B* and *GMR-grim* flies were obtained from the lab of IK Hariharan; *w*; *UAS R406W *and *w; Tubgal4* (FlyBase ID = FBti0012687) and *UAS green fluorescent protein (GFP)* (FlyBase ID FBti0012686) elements were obtained from the Bloomington Stock Center.

### 2.2. R406W Tau Experiments and Visual Scoring of Eye Abnormality

*eygal4/SM6-TM6B* flies were crossed to *UAS R406W* flies to generate *ey *>* R406W *flies. *ey *>* R406W* eggs were laid in and reared on instant fly medium formula 4–24 supplemented with 2.8 *μ*g/mL GSPE (“GSPE food”), the concentration used in our previous study [13], or control food supplemented with an equivalent volume of water (GSPE solvent, vehicle control). 

Flies of the indicated genotypes were examined side by side under a dissecting scope. Eye regions which formed no ommatidia (therefore lacked eye tissue completely) were considered “0 = no eye” and required no comparison. For eye areas which did form obvious ommatidia/eye tissue, wild-type control flies were examined at the same time to establish the “4 = almost wild-type” eye size, shape, and pattern upper limit. Eyes that formed but did not reach the “4 = almost wild-type” category were lined up under the scope by size and grouped into categories based on the amount of eye tissue present. Because eyes were grouped side by side under the microscope, relative sizes were easy to establish. When additional conditions or repeated experiments were examined, representative eyes of each category from the previous experiments were examined again in parallel to ensure that the same designations/scoring were maintained.

### 2.3. Statistical Analyses

The distribution of eye sizes was reviewed and found to have negative kurtosis, indicating a distribution that was more uniform over the range from 0 to 4 than a normal distribution. Negative kurtosis does not substantially impair analysis of variance.

 In addition to overall analysis of variance (with trials nested within gender and crossed by treatment), separate *t*-tests were performed for each trial tested.

## 3. Results

### 3.1. GSPE Treatment Improves the Eye Phenotype of ey > R406W Flies

**Figure 1:**
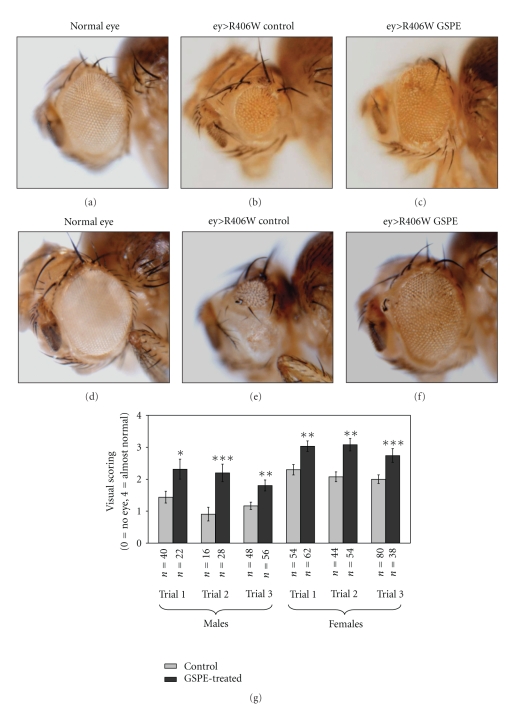
*GSPE attenuates R406W tau overexpression in the fly eye*. Male and female eye size differs; male eyes are shown in (a)–(c), and female eyes are shown in (d)–(f). Overexpression of *R406W* early in eye development results in a small or no eye ((b), (e)). Eyes that do not overexpress *R406W* are shown in (a), (d) for comparison. GSPE treatment ameliorates the reduction in eye size (representative eyes shown in (c), (f)). GSPE treatment does not affect normal-eye development (not shown). (g) The range of *ey *>* R406W *phenotypes varies between trials, so treatment comparisons were made within experiments. The average visual score (0 = no eye, 4 = almost normal eye) ± SEM of male and female eyes is shown for three independent trials for males and three independent trials for females. The number of flies (*n*) and *P* value calculated using a paired *t*-test on GraphPad online software are indicated beneath each trial. Flies were collected within 5 days of eclosion. Statistically significant improvement (*P* < .05) was observed in multiple independent experiments. * indicates *P* < .05, ** indicates *P* < .01, and *** indicates *P* < .001. (An excerpt of these images has been presented without data in a review manuscript by the author.)

*ey *>* R406W* flies show a range of phenotypes from no eye to small, abnormal eyes. The reduced size and abnormal morphology of *ey *>* R406W *eyes were improved by GSPE (F(1,531) = 57.29; *P* < .0005). The incidence of the worst outcomes (visual score of 0 or 1) decreased upon GSPE treatment in each trial while the incidence of the best outcomes (visual score of 3 or 4) increased upon GSPE treatment in each trial. There were also differences in the size of male and female eyes (F(1,531) = 58.62; *P* < .0005). Representative eyes are shown in Figures 1(a)–1(f) ((a)–(c) male; (d)–(f) female). Nonetheless, there was no interaction between gender and treatment (F(1,531) = 0.30; *P* = .59); the difference between treated and untreated flies was similar in male and female flies. Separate *t*-tests showed that each of the six trials had a statistical difference with GSPE treatment characterized by increased visual scoring relative to untreated controls (Figure 1(g)). However, within the combinations of gender and treatment, the trials did not differ significantly (F(4,531) = 1.90; *P* < .11).

### 3.2. GSPE Effects on Gal4/UAS-Mediated Protein Expression

**Figure 2:**
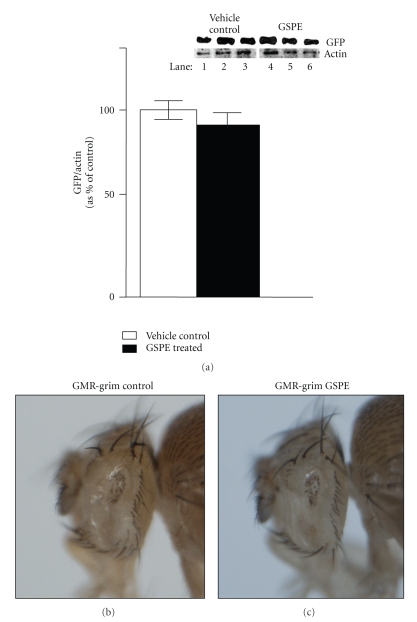
*GSPE does not inhibit Gal4-mediated expression of GFP*. (a) Western-blot analysis of lysates from *TubGal4 *>* GFP* flies (created by crossing *TubGal4 *flies to *UAS-GFP* flies) reared from egg deposition either on food supplemented with water (vehicle control) (Lanes 1–3) or reared on GSPE food (lanes 4–6). Each lysate was prepared from 6 fly heads, and three independent groups of 6 flies per group (lane) are shown. The same immunoblot was probed first with antibodies raised against GFP (upper panel) and then immunoblots were stripped and reprobed with antiactin antibodies (lower panel). (b) *GMR-grim* flies have very small, almost absent eyes when reared on control food. (c) Eye size is not affected when *GMR-grim* flies are reared on GSPE food.

To address the concern that GSPE could disrupt the production of the toxic proteins by the Gal4/UAS system, we performed control experiments using the Gal4/UAS system to determine whether GSPE treatment prevents expression of GFP using a *UAS-GFP* transgene and *TubGal4* (constitutively expressed as Gal4). We found no detectable alteration in the production of GFP both by fluorescent imaging of GFP (not shown) and by Western analysis (Figure 2(a)).

### 3.3. GSPE Does Not Affect the Small-Eye Phenotype Caused by Overexpression of the Proapoptotic Gene Grim

To investigate if GSPE acts on cell death pathways directly, we examined the effects of GSPE treatment on flies overexpressing the pro-apoptotic gene *grim* in differentiating cells in the eye (which causes a very small eye). *GMR-grim* flies (which overexpress the pro-apoptotic gene *grim* in differentiating cells in the eye) laid eggs on control food or GSPE food. *GMR-grim *flies reared entirely on GSPE food showed no effect of reduced eye size (Figures 2(b)-2(c)). 

## 4. Discussion

We have demonstrated that GSPE treatment benefits a distinct *Drosophila* model of neurodegeneration involving aggregation *in vivo *(Figures 1 and 2). GSPE showed no effect on Gal4-mediated expression of GFP (Figure 2(a)), indicating that GSPE does not block Gal4/UAS-dependent gene expression. GSPE had no effect on eye size in *GMR-grim* flies (which overexpress in the eye the pro-apoptotic gene *grim* which activates caspases and severely reduces eye size [14]) (Figures 2(b)-2(c)). Thus, although the mechanism remains unknown, GSPE-mediated improvements in the *Drosophila* eye model likely occur subsequent to production of the toxic protein but upstream of caspase activation. 

Presently there is no treatment for the devastating progressive neurodegenerative disorders involving tau abnormalities. Continuing investigations suggest that oligomeric forms of these misfolded proteins may play a seminal role in disease etiology. Results from our studies show that GSPE may rescue abnormal tau phenotype in a Drosophila model of tauopathy. This study is consistent with our previous study, which provided evidence that GSPE could inhibit tau peptide aggregation [12] and prevent oligomerization of A*β*  peptides into high molecular weight species in models of AD-amyloid neuropathology [10, 11]. The study supports further investigations of GSPE in models of tauopathies, for example, frontotemporal dementia and CBD, among others.
